# An Account of the Ringwood Cases

**Published:** 1808-03

**Authors:** 


Medical and Phyſical Journal.

VOL. XIX.] MARCH, 1808. [NO. 109.

*Printed for R. PHILLIPS, by W. Thorne, Red Lion Court, Fleet Street, London*

### *To the Editors of the Medical and Physical Journal.*

GENTLEMEN,

THE melancholy circumstances which have recently occurred at Ringwood, in Hampshire, have been related to the world in most of the public papers, and in some other of the common channels of intelligence. While the report of the failures of Vaccination was confined to such vehicles, it was not of sufficient importance to deserve a formal reply; yet as an alarming pamphlet has appeared, entitled "The Fatal Effects of Cow-pox Protection," and as the public attention must necessarily be excited by a subject which involves the dearest interests of this nation and the world at large, it is a duty incumbent on every man, who is in possession of facts that are calculated to remove the misapprehensions which erroneous reports may occasion, to give those facts all possible publicity. And though your Journal has of late been properly shut against indiscriminate vaccine intelligence, the acknowledged importance of this investigation will doubtless induce you to grant an immediate insertion to the following remarks on the pamphlet before alluded to.

The Author seems desirous to evince his genius in the commencement of his labours. His title page contains a notorious *Bull,* which may serve as a frontispiece to his work. Perhaps, as he was writing on the *Cow*-pox, he conceived such an ornament would not be unappropriate. He calls his pamphlet, "*The Fatal Effects of Cow-pox Protection.*" He immediately after tells us, that "*the Cow-pox affords neither security nor protection;*" so that, according to his own account, he has written a book to shew the fatal effects of *a Non-entity.*

He then acknowledges his expectation to be ranked among those, whom the College of Physicians represent as "*ignorant and designing men.*" There is indeed a degree of pleasing candour in this avowal; for, to acknowledge one's folly is the first step to wisdom. I would however simply ask an unprejudiced observer, what degree of credit is to be attached to the publications of a man, whom, by his own confession, the first medical body in the world accuses *of gross ignorance and wilful misrepresentation.*

The Author congratulates himself in the hope that the concealment of his name will prevent his expulson from the banquets of Warwick-lane. I am sorry that I cannot flatter him in this hope. *Ubicunque est diu latere non potest.* A reader of no extraordinary discernment must perceive, that the "*Fatal Effects,*" and "*Serious Reasons,*" were the bantlings of the same sire. Ignorance and misrepresentation will invariably betray their author.

He calls vaccine inoculation a *desperate attempt.* It is some consolation however to the friends of vaccination to reflect, that this *desperate attempt* has nearly annihilated the small-pox in our East Indian settlements, New Hampshire, and New Portsmouth; in Vienna, Geneva, Berlin, and Marseilles, and in many districts of Great Britain. That this *desperate attempt,* according to the College Report, holds forth the prospect of exterminating the small-pox from the face of the earth; and that this *desperate attempt* has been sanctioned by the British Parliament, by all the European governments, and by every civilized nation in the world.

It is not surprizing that the anti-vaccinists should object to the remuneration of Dr. Jenner. Small-pox inoculation has long been a considerable source of medical income. It was natural, therefore, that the anti-vaccinists should be particularly chagrined, to see the justice of government increase the revenue of that man, who, by mitigating the small-pox, has so materially diminished their revenue. Grave-diggers and undertakers will no doubt most cordially unite in the discontent expressed by the Author of the "Fatal Effects."

He talks of the sum, for which Dr. Jenner's *modest request petitioned.* Now it must be notorious to every man, who has paid the smallest attention to the subject, that the second remuneration of Dr. Jenner did not originate with any petition or solicitation of any kind whatsoever on Dr. Jenner's part, but was altogether the free and spontaneous act of our legislature, primarily proposed by Lord Henry Petty, and completed by the motion of Mr. Perceval, with only one dissentient voice. He

He complains that this petition (though, as I observed before, there was *no* petition,) was debated, "*quite at the end of the session, when the tired members had retreated to their country residences.*" Let him turn to the Parliamentary Journals, and he will find that a sum vastly larger than this was voted in the same week. To this vote however he makes no objection.

With respect to the parliamentary evidence on vaccination in 1802, I shall simply observe, that, to use our Author's own words, "*it was dignified by Princes and Peers, and supported by Physicians, Surgeons, and Clergymen.*" It was opposed by Dr. Moseley, Dr. Rowley, and Mr. Birch.

The Author exults in the idea that the vox populi in the metropolis is averse to vaccination. Whether this be a subject for exultation or regret, let the bills of mortality declare. In the year 1804, when vaccination was more generally practised, only 622 died of the small-pox. In the last six months, we are told, the practice of vaccination has decreased, and that of inoculation has prevailed. And what has been the result? In the course of four weeks 290 miserable victims were hurried to an untimely grave, within the bills of mortality alone, by the *mild* and *gentle* small-pox.

The Author deprecates the idea, that coercive vaccination should deprive the poor of *the blessings of inoculation.* The generality of readers may perhaps be at a loss to know what can be the *blessings* of keeping up the small-pox by inoculation, when we have the power of totally preventing it. The Author, therefore, has kindly informed us, in his "*Serious Reasons for uniformly opposing the Practice of Vaccination.*" "Why is it not remembered, that in the populous part of the metropolis, where the abundance of children surpasses the means of procuring food and raiment for them, this pestilential disease is considered as a *merciful provision on the part of Providence to lessen the burthen of a poor man's family!!!*

The Author lays peculiar emphasis on two instances of ulceration which followed vaccination. Did he never hear of ulcerations following variolous *inoculation?* I must beg leave to recall to his memory a circumstance, which he cannot for a moment hesitate to acknowledge. At the very time that he appeared before a Committee of the House of Commons, to give his evidence against vaccination, and asserted that the inoculated small-pox in his hands was never attended by dangerous consequences, had he not under his care a child whom *he* had recently inoculated for the small-pox, and who, in consequence of the inoculation, was afflicted by a most dangerous abscess in the thigh? Our sagacious Author, who, I presume, had never heard of ulcerations following the small-pox, trembled for the safety of the child, and deemed it indispensably requisite to call in Mr. Cline. If our Author has forgotten this circumstance, let me remind him that the residence of his patient was not many miles distant from Grosvenor Place.

Particular pains are taken to collect the various cases in which vaccination is said to have failed. How is it, then, that we have not been favoured with any instances, in which those who were vaccinated by Dr. Jenner have subsequently undergone the small-pox? Dr. Jenner has vaccinated many thousands, and his inoculations have proceeded in an uninterrupted series for nearly ten years; yet who has ever produced a case in which his vaccination failed? Can there be a more convincing and decisive proof, that when the small-pox is communicated to a person, in whose arm the vaccine matter has been inserted, the failure arises from the ignorance or carelessness of the inoculator, or the patient? If one man vaccinates his thousands, without a single anomalous instance, and another vaccinates a few, and finds among them many failures, no argument can be wanting to explain the difference. The operations of Nature are uniform; but the ignorance of man may produce deviations in all her works.

But the principal strength of the "Fatal Effects," consists in the disasters of Ringwood. This subject will therefore claim our principal attention. I shall not answer the Author in a rhapsody as diffuse and incoherent as his own. Plain truth may be told in a few words. The Author published a pompous account of the failures of vaccination, in the Morning Post, of January 1st. He *then* wrote to Mr. Westcott, an eminent surgeon, of Ringwood, to enquire whether the account was true!!! This is a genuine instance of anti-vaccine candour and veracity. Mr. Westcott, in a letter to Mr. Blair, declared that the statement was "*directly wrong*, and that its Author *has asserted a falsehood.*" A medical deputation from the Royal Jennerian Society, proceeded to Ringwood, and instituted a minute investigation into the subject. The result of their enquiry has since been given to the world, of which the following is an extract:

"The small-pox appeared at Ringwood, about the middle dle of September, and rapidly spread through the town and neighbourhood. Vaccine inoculation did not commence till the 23d of October. It is therefore evident, that all those persons who were vaccinated, had been previously exposed to the contagion of small-pox. Some of these persons had the small-pox, at the same time with the cow-pox, in consequence of previous infection. In others, vaccine inoculation did not take effect; consequently they were not rendered insusceptible to the infection of the small-pox. In several instances, dry cow-pock matter was dissolved in water, almost boiling, previous to insertion; and on this account it frequently failed to produce any effect. Above 200 persons, however, have been successfully vaccinated, and have been protected from the small-pox, though much exposed to its infection in various ways.

"It was reported that several persons at Ringwood, who were inoculated with the cow-pock some years ago, lately had the small-pox: but no satisfactory evidence was given to establish the fact, as it appeared either that their arms had not been inspected by the inoculators after vaccination, or that there was no proper scar left behind. It was insiduously reported also, that two persons died of the cow-pock, or, as it has been termed, "*the Vaccine Ulcer:*" but it is positively asserted by the surgeons who inoculated them, that no vaccine ulcer nor cow-pock took place in either of those instances, and that the patients died of other diseases; one of them of *an apoplexy.*"

Mr. Westcott, and Mr. Macilwain, two resident surgeons, and Dr. Fowler, an eminent physician of Salisbury, unite in declaring their firm opinion, "*that not one person in Ringwood, or its vicinity, caught or had the small-pox, after going through complete and regular vaccination.*" And so fully has the whole affair been investigated to the satisfaction of the inhabitants of Ringwood, and the surrounding country, that many persons, who before opposed vaccination, are now become strenuous supporters of it.

In the 43d page, Dr. Jenner is summoned to declare, "*what was the reason that the Gloucester Regiment, vaccinated with matter, sent by himself, were not secured from subsequent small-pox.* The reply to this query is plain and easy. An application was made both to the colonel and surgeon of the regiment, for information on the subject, who declare, that *in no instance, either in or out of the regiment, have they known vaccination fail.*

We are told, that the public prints "are by some means

 shut shut against all information, which militates against the prevailing bias, in favour of vaccination." How then did the author contrive to procure the insertion of the before-mentioned paragraph, written by himself in the Morning Post, respecting the Ringwood cases? Perhaps he may tell us in reply, that these citadels of public intelligence are shut against ordinary mortals, but that he, like Jupiter, *entered them in a golden shower.*

The observation, that *it is improper to contaminate children with the grease of a horse's heel*, is too puerile, too unphilosophical, and too vulgar to merit any reply.

The author tells us, that " the public has been deceived, *experimented*, and miserably *disastered*." This observation shews that he understands the English language, as well as he understands vaccination and medicine.

I sincerely congratulate Mr. Ring and Dr. Thornton, on the high compliment paid them in the 45th page. We are informed that these gentlemen, in ignorance and abuse, are equal to the College of Physicians!

The author promises us in his motto, that " *he will a round, unvarnished tale deliver.*" This *delivery*, however, is similar in nature and effect to many of his former *deliveries.*

Parturiunt montes,—nascetur ridiculus mus.

The Report of the Royal College of Physicians is continually quoted, misquoted, and censured. This Report proceeds from too able and too illustrious a body, to require support from the pen of an individual. Profound as is the veneration I must entertain for the professional talent of the author of the " Fatal Effects," I trust that gentleman will pardon me, if I candidly confess, that I, like the public in general, have been absurd enough to take up an opinion, that a dissertation on a medical subject, proceeding from the Royal Colleges of Physicians and Surgeons of London, Edinburgh, and Dublin, is almost as worthy of universal confidence, as the publications of —our author himself. I am, &c.

*February*, 1808. COSMOPOLITOS.

